# The Effect of Y Addition on Oxidation Resistance of Bulk W-Cr Alloys

**DOI:** 10.3390/ma17235749

**Published:** 2024-11-24

**Authors:** Anicha Reuban, Jie Chen, Ivan Povstugar, Andrey Litnovsky, Jan Willem Coenen, Christian Linsmeier, Jesus Gonzalez-Julian

**Affiliations:** 1 Institute of Fusion Energy and Nuclear Waste Management–Plasma Physics (IFN-1), Forschungszentrum Jülich GmbH, 52425 Jülich, Germany; ji.chen@fz-juelich.de (J.C.); a.litnovsky@fz-juelich.de (A.L.); j.w.coenen@fz-juelich.de (J.W.C.); ch.linsmeier@fz-juelich.de (C.L.); 2 Institute of Mineral Engineering (GHI), RWTH Aachen University, 52074 Aachen, Germany; gonzalez@ghi.rwth-aachen.de; 3 Ernst Ruska-Centre for Microscopy and Spectroscopy with Electrons (ER-C), Forschungszentrum Jülich GmbH, 52425 Jülich, Germany; 4 Institute of Energy Materials and Devices–Materials Synthesis and Processing (IMD-2), Forschungszentrum Jülich GmbH, 52425 Jülich, Germany

**Keywords:** self-passivating W alloys, high-temperature oxidation, chromia, reactive element effect, yttrium

## Abstract

The self-passivating tungsten-based alloy W-11.4Cr-0.6Y (in wt.%) is a potential plasma-facing material for the first wall of future fusion reactors, which has been shown to suppress oxidation of tungsten and withstand temperatures of up to 1000 °C. In this study, the effect of Y addition on the microstructure and oxidation behavior of W-11.4Cr alloy at 1000 °C is analyzed by comparing it with W-11.4Cr-0.6Y, both prepared using identical synthesis routes. While the binary W-Cr alloy already exhibits improved oxidation resistance over pure W due to the formation of an outer Cr_2_WO_6_ layer, it still shows a tendency for spallation and, hence, is not protective. A continuous passivating chromia layer is only obtained with the addition of Y, and we demonstrate that it results in a 50-fold decrease in the oxide growth rate and eliminates the preferred growth of the oxide at edges seen in the binary alloy. Although a porous, complex oxide scale containing mixed oxide layers and WO_3_ is formed in both cases, the addition of Y results in lower porosity, which makes the oxide scale more adherent.

## 1. Introduction

Tungsten (W), being the metal with the highest melting point [1], shows great potential for use in high-temperature applications. This includes applications such as the first wall of a fusion reactor, also due to other favorable properties such as high thermal conductivity, resistance to erosion under plasma operation, and low tritium retention [2,3]. However, one of the major disadvantages of pure W is its oxidation behavior, which becomes important to consider from a safety perspective. For example, in the event of a loss-of-coolant accident (LOCA) with air ingress, temperatures can rise up to 1100 °C [4], thereby creating conditions for oxidation of W into WO_3_, which can readily sublimate at these temperatures at the rate of hundreds of kg per week [5]. Neutron irradiation of the first wall of the fusion reactor activates W, and WO_3_ formed and released into the environment will be radioactive. To improve the oxidation resistance and prevent WO_3_ release, W-based alloys containing the addition of passive oxide-forming elements, such as Si and Cr, sometimes with a combination of Ti and Nb, have been designed and explored [6,7,8,9,10]. However, there are some limitations in the selection of alloying elements. Despite the high oxidation resistance of alumina formers, the usage of Al in materials for fusion reactor applications must be avoided since it can transmute into radioactive products with long half-lives [3]. It is also better to avoid the use of Si due to the formation of brittle tungsten silicides, which degrade the workability of W [11]. Hence, the focus was shifted to the development of W-Cr-based systems. 

In binary W-Cr alloys, the presence of Cr alone is not enough to create a fully passivating Cr_2_O_3_ layer, as seen in the study of binary W-Cr alloys of varying compositions, especially at temperatures above 800 °C [7]. The addition of elements such as Y and Zr to the W-Cr system, even at low amounts, has been shown to drastically increase the oxidation resistance [5,12,13], which is attributed to the so-called reactive element effect [14]. For example, studies on thin films of W-Cr and W-Cr-Y alloys have shown much better performance for the ternary alloy with an addition of merely 0.6 wt.% Y [5].

The reactive element effect has been extensively studied for Y in many materials such as Ni-Cr-, Fe-Cr-, Co-Cr-, and Fe-Cr-Al-based alloys [15,16,17,18,19,20,21,22]. Several mechanisms have been proposed, the most significant of them being the effect of Y segregation at oxide grain boundaries (GBs), which can affect the diffusion of the cations (Al or Cr ions for alumina or chromia formers, respectively), as well as anions (O ions) involved in the formation of the oxide scale. It has been shown that the oxide growth direction changes from the outward direction to the inward direction with the addition of Y, which is more beneficial since it reduces the possibility of void formation at the oxide–alloy interface, hence increasing the adherence of the oxide to the substrate [23]. However, studies of the Y effect in W-based alloys are very limited. Studies comparing bulk W-Cr and W-Cr-Y alloys produced by hot isostatic pressing (HIP) have been performed, but the amount of Cr is different for the binary and ternary alloys [12,24]. Additionally, although the oxidation rates were compared, the oxide scale microstructures with and without Y were not analyzed. A direct comparison of the effect of Y addition on the oxidation rate and oxide scale microstructure has been conducted on W-Cr thin films [5]; however, thin films have a different microstructure as compared to bulk alloys, as well as limited supply of Cr and Y, due to the limited volume of material. Therefore, they cannot accurately represent the oxidation behavior of bulk alloys. Since reactive elements are also commonly added as oxide dispersions, the addition of Y_2_O_3_ to W-Cr is also expected to have a similar effect as the addition of Y, and a study comparing the oxidation rates of W-Cr and W-Cr-Y_2_O_3_ has been performed before [25]. A recent study has shown that the addition of Y_2_O_3_ results in the formation of additional oxide dispersoids and results in better oxidation resistance but lower fracture toughness than W-Cr-Y [26].

The main focus of this work is to investigate how the addition of Y influences oxidation resistance by studying the oxidation behavior of two bulk-sintered alloys—the binary W-11.4Cr alloy and the ternary W-11.4Cr-0.6Y alloy. The amount of Cr and the preparation route of both alloys were kept the same, with the only difference being the addition of 0.6 wt.% of Y in the ternary alloy. These compositions were chosen based on the work conducted previously on thin films, where the optimum concentrations of Cr and Y were determined [5]. The optimization of Y concentration was also performed on bulk sintered alloys, where the composition W-11.4Cr-0.6Y was shown to have the best oxidation resistance [27]. The two alloys were oxidized under the same conditions, and the differences in oxidation kinetics and behavior at the microstructural scale were analyzed, enabling us to infer the effect of Y on oxidation behavior.

## 2. Materials and Methods

The binary W-11.4Cr and ternary W-11.4Cr-0.6Y (in wt.%) alloys were prepared by mechanical alloying of elemental powders, followed by consolidation using the field-assisted sintering technique/spark plasma sintering (FAST/SPS). Elemental powders of tungsten (average particle size, APS 4 µm, 99.9% pure), chromium (APS 45 µm, 99.7% pure), and yttrium (APS 500 µm, 99.9% pure) were mixed in the desired ratio (68.9W-31.1Cr in at.% for the binary and 67.9W-31.1Cr-1.0Y in at.% for the ternary). The powder mixtures were milled in a planetary ball mill (Retsch PM400 MA, Retsch GmbH, Haan, Germany) for 60 h at 198 rpm under an Ar atmosphere. Tungsten carbide milling balls were used, and the inner wall of the milling jar was coated with tungsten carbide as well. The ball-to-powder ratio was 5:1. After ball milling, 25 g of the mechanically alloyed powder was placed into a graphite mold with a diameter of 20 mm, and a hydraulic press was used to create a green compact. This was then consolidated using the FAST/SPS facility (FCT-HPD5, FCT Systeme GmbH, Frankenblick, Germany) under vacuum and the application of a uniaxial pressure of 50 MPa throughout the process. It was heated up at a rate of 200 K/min until a maximum temperature of 1460 ℃, with no isothermal holding time at this temperature. These are the optimized sintering parameters for the synthesis of the ternary W-Cr-Y alloy according to [28], and in the present study, the same parameters were used for the synthesis of the binary W-Cr alloy as well, so that the only differing factor in both these alloys is the addition of 0.6 wt.% Y. The resulting ingots are well-densified, having a relative density of over 98%, which is measured using the Archimedes principle.

Samples for oxidation testing were cut from the sintered ingots using electrical discharge machining (EDM). For thermogravimetric analysis, the samples were cut into cuboidal shapes of dimensions approximately 5 mm × 4.5 mm × 3 mm so that the total surface area of the sample is approximately 1 cm^2^. For the oxidation performed under a static atmosphere in a tube furnace, flat samples of dimensions 10 mm × 10 mm × 1 mm were used. The samples in all cases were ground to a 1200-grit surface finish before oxidation was performed. 

Oxidation along with simultaneous thermogravimetric analysis (TGA) was performed using the thermogravimetric analyzer TAG-16/18 (from Setaram Inc., Caluire-et-Cuire, France) at the ThermoLab [29]. The samples were isothermally oxidized at a temperature of 1000 °C for 20 h in synthetic air with 70% relative humidity (at 25 °C). They were heated at a rate of 10 K/min in an argon atmosphere, with two slower ramps of 5 K/min and 2 K/min used in the end to avoid overshooting the set temperature, and then humid synthetic air was introduced. After the exposure was completed, the heating chambers were flushed with argon and cooled in three temperature ramps of 40 K/min, 20 K/min, and 10 K/min. 

Samples were also oxidized separately in a tube furnace. The furnace has an integrated air-tight silica tube, long enough to have well-defined hot and cold zones. A mixture of Ar-20%O_2_ was introduced into the furnace and heated to 1000 °C, after which the samples were moved from the cold to the hot zone and kept there for 8 h. After the desired exposure time, the samples were moved back to the cold zone and allowed to cool down. 

X-ray diffraction (XRD) using Cu Kα radiation was performed on the samples before and after oxidation using the D8 Discover from Bruker AXS GmbH, Karlsruhe, Germany, over the 2θ range 10-120°, with a step size of 0.02° and 2 s as the time per step. The Inorganic Crystal Structure Database (ICSD) [30] was used for phase indexing. The surface and cross-sectional microstructures were characterized using the ZEISS Crossbeam XB 540 FIB-SEM (field emission scanning electron microscope with focused ion beam) manufactured by Carl Zeiss Microscopy GmbH, Oberkochen, Germany. The focused ion beam consisting of Ga^+^ ions was used to mill the cross-section of the oxide scale, and the SEM was used to obtain secondary electron images. ImageJ software (version 1.53k) [31] was used to calculate the grain size based on the linear intercept method, as well as obtain the areal particle density from the SEM images. A corresponding elemental analysis was performed in the FIB-SEM tool using the in-built energy-dispersive X-ray spectroscopy (EDX) detector from Oxford Instruments plc, Abingdon, England. An accelerating voltage of 5 kV was used for imaging, while 12 kV was used for the EDX analysis. The following characteristic X-ray lines were used for analysis: W Mα, Cr Kα, Y Lα, and O Kα.

## 3. Results

### 3.1. Microstructure of As-Sintered Alloys

**Figure 1:**
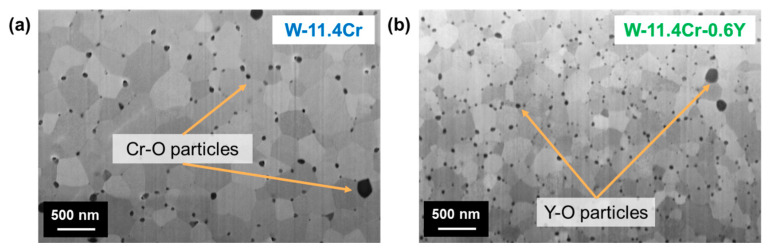
SEM images of the as-sintered microstructure of (**a**) the binary alloy W-11.4Cr and (**b**) the ternary alloy W-11.4Cr-0.6Y.

In the as-sintered state, as shown in Figure 1, both the binary and the ternary alloys consist of the W-rich phase (αW, Cr). In both alloys, the GBs are decorated with oxide particles—Cr-O particles in the case of the binary and Y-O particles in the ternary alloy. Although Y_2_O_3_ is the stable form of the oxide, it is not known if these particles are stoichiometric or possibly contain Cr since the composition of the particles has not been determined in this study. The areal density of these particles also varies: the density of Cr-O particles in the binary W-Cr system is ~9 particles/µm^2^, being much lower than that of the Y-O particles in the ternary W-Cr-Y, which is ~29 particles/µm^2^. Another key difference in the microstructure is the grain size, which is 300–400 nm in the binary but only ~180 nm big in the ternary alloy.

### 3.2. Oxidation Behavior and Kinetics

**Figure 2:**
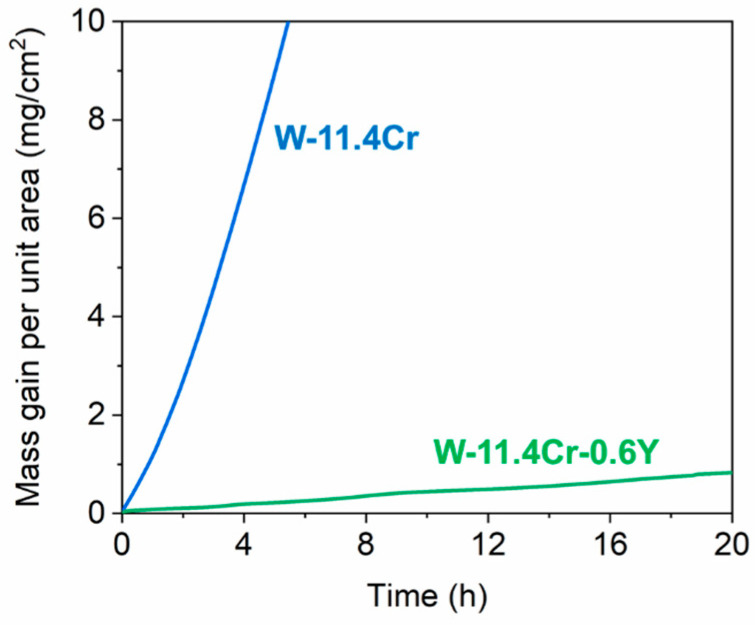
Mass gain curves of the binary (blue) and ternary (green) alloys oxidized at 1000 °C.

Figure 2 shows thermogravimetric analysis plots obtained from the isothermal oxidation of the binary and ternary alloys at 1000 °C in a synthetic air atmosphere with 70% relative humidity at 25 °C. The mass gain of the binary alloy rapidly increases with time, whereas the ternary alloy shows a much slower rate of oxidation, with the mass gain being ~0.8 mg/cm^2^ even after 20 h. By having a much slower rate of oxidation and no indication of spallation, the ternary alloy seems to show a better oxidation resistance behavior.

In order to quantify the rate of oxidation, the data were first fit to a power law equation of the general form.

Δm = k · t^n^ (1)

Here, Δm is the mass gain per unit area in mg/cm^2^, and t is the time in seconds. The scale growth exponent n determines the type of oxidation kinetics. As a general rule, the lower the value of n, the slower and, hence, more oxidation-resistant the material is [32]. The value of the growth exponent n is 1.27 for the binary and 0.92 for the ternary, indicating that in this time scale, both the alloys show close to linear oxidation kinetics, with the binary showing even faster than linear kinetics. The linear growth rate of the binary alloy is 5.64 × 10^−4^ mg·cm^−2^·s^−1^ and is 1.1 × 10^−5^ mg·cm^−2^·s^−1^ for the ternary alloy. Hence, the addition of Y results in an apparent 50-fold decrease in the oxide growth rate as compared to the binary alloy.

### 3.3. Morphology and Microstructure of the Oxide Scale

**Figure 3:**
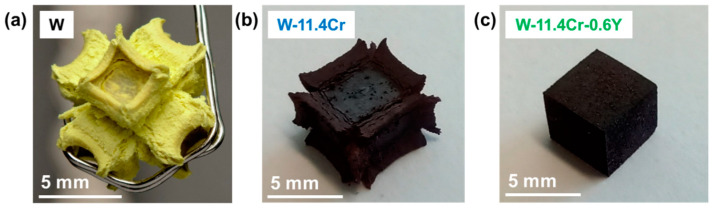
Cuboidal samples oxidized at 1000 °C of (**a**) pure W for 10 h (reprinted from [34] with permission from Elsevier), (**b**) W-Cr for 20 h, and (**c**) W-Cr-Y for 20 h.

In addition to the TGA curves, the improved oxidation resistance with the addition of Cr and Y to tungsten is also quite evident when looking at the morphology of bulk samples after they are oxidized under the same conditions. Figure 3 shows the comparison between cuboidal samples of pure W, W-Cr, and W-Cr-Y, all oxidized at 1000 °C. We can see that the oxide scale formed on W, having a Pilling–Bedworth ratio of 3.4 [33], cannot serve a protective function since the oxide rapidly grows and expands into a volume more than three times that of the metal it is formed from. The binary alloy shows an improvement over pure W, but the amount of oxide formed is still large, especially at the sample edges, and shows there is a tendency for spallation. On the other hand, the ternary alloy still retains the original cuboidal geometry after oxidation, indicating that the formed oxide layer is slow-growing and dense. 

**Figure 4:**
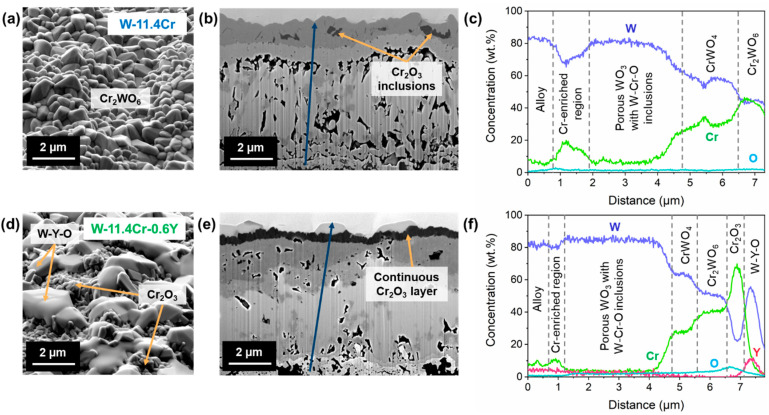
SEM images of the oxidized binary W-Cr alloy: (**a**) the surface, (**b**) cross-section of the oxide layer, and (**c**) EDX line scan showing the elemental concentrations along the dark blue arrow in (**b**); SEM images of the oxidized ternary W-Cr-Y alloy: (**d**) the surface, (**e**) cross-section of the oxide layer, and (**f**) EDX line scan showing the elemental concentrations along the dark blue arrow in (**e**).

**Figure 5:**
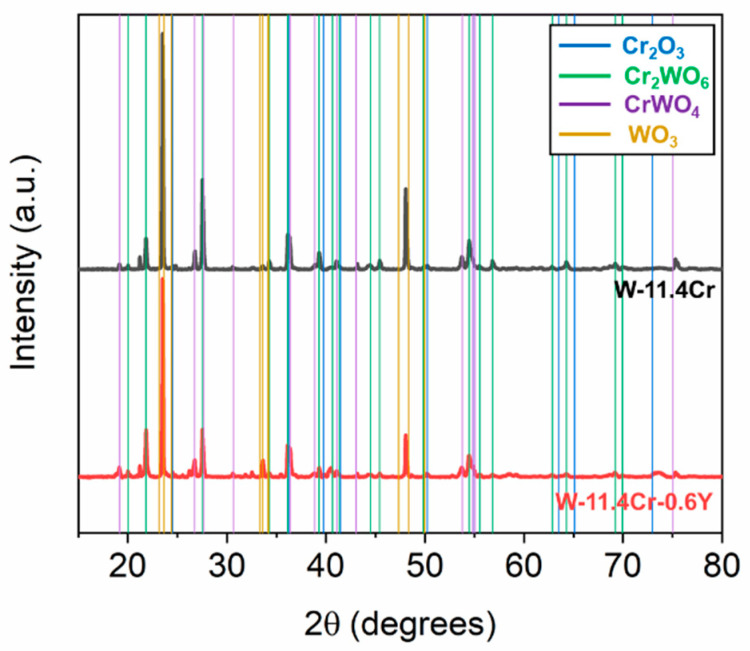
XRD patterns of the binary W-Cr and ternary W-Cr-Y alloys oxidized for 8 h at 1000 °C in dry air. The overlay lines show the major peak positions of chromia, mixed oxides, and tungsten oxide based on [36,37,38,39].

Apart from the overall morphology, there are also several differences in the microstructure of the oxide scales on the binary and ternary alloys. Figure 4 shows a comparison between the surface and cross-sectional microstructures of the oxide scale after 8 h of isothermal oxidation at 1000 °C in dry air. In both cases, a complex oxide scale consisting of a number of different oxides with varying compositions is observed. The phases present were determined based on the XRD patterns, shown in Figure 5, and the location of each phase in the oxide scale was determined with the help of the elemental distribution obtained through EDX. The surface of the oxidized binary alloy, shown in Figure 4a, consists of the mixed Cr_2_WO_6_ oxide about 1 µm thick, containing only some small inclusions of Cr_2_O_3_, which can be seen in the cross-section in Figure 4b. In contrast, a continuous protective Cr_2_O_3_ layer of ~400 nm thickness is formed on the ternary alloy, along with large particles of a mixed W- and Y-containing oxide present on the surface, as seen in Figure 4d,e. Below the outermost layer, a porous mixed oxide region is found in both alloys, with higher porosity in the case of the binary alloy. The formation of pores is due to the Kirkendall effect [35]. However, the number and composition of the distinct oxide layers present in this region vary. The oxide scale in the binary alloy consists of an outermost layer of Cr_2_WO_6_, followed subsequently by CrWO_4_, a porous WO_3_ region with some mixed W-Cr-O inclusions, and a Cr-enriched oxide region close to the interface with the alloy. The oxide scale in the ternary alloy has more layers but follows a similar pattern. Below the continuous chromia layer, we see Cr_2_WO_6_ and CrWO_4_ layers, WO_3_ containing mixed oxide inclusions, and again a Cr-enriched oxide region. In general, starting from the outermost layer, the Cr content gradually decreases from layer to layer before rising once again near the oxide–alloy interface, as can be seen from the elemental concentrations across the cross-section obtained through EDX, shown in Figure 4c,f. In addition, there is also some internal oxidation that takes place in both alloys, resulting in the formation of chromia within the alloy, close to the alloy–oxide interface (see Supplementary data for lower magnification images of the cross-section).

## 4. Discussion

In the as-sintered state of the ternary W-Cr-Y alloy, Y is found in the form of oxide particles decorating the alloy GBs. The density of these Y-O particles is ~3 times that of the corresponding Cr-O particles in the binary alloy. Due to the GB pinning effect [40] of the large number of Y-O particles, the grain growth is restricted, which results in a finer grain size as compared to the binary W-Cr alloy. A finer grain size corresponds to a larger available GB area and is a beneficial aspect for oxidation resistance when the Cr diffusion needed to form the protective oxide layer proceeds primarily via alloy GBs. Improved oxidation performance with smaller grain size has been observed before in the W-Cr-Y system, attributed to an enhancement in overall Cr transport via the alloy GBs to form the protective Cr_2_O_3_ layer [28].

After oxidation, the formation of a complex mixed oxide scale occurs in both the binary and the ternary alloys. In previously published studies of the bulk ternary alloy W-11.4Cr-0.6Y, with a grain size of ~200 nm and less, oxidation was shown to result in the formation of a continuous chromia layer, along with internal oxidation, with no mixed oxide formation even after oxidation times of up to 44 h [28]. In the present study, we analyzed six different batches of the ternary W-Cr-Y alloy having an identical composition and observed that mixed oxide formation indeed occurs in samples from all those batches, both in dry and wet atmospheres (details can be found in the Supplementary data). Formation of a complex scale with mixed oxides is much more common than the formation of a single-layer Cr_2_O_3_ scale, which was observed only in a few early experiments in two batches and could not be reproduced even with the identical composition, similar grain size, Y-O particle distribution, and Cr content in the as-sintered state (as measured by EDX). Therefore, the former microstructure showing mixed oxides seems to be a typical scenario for the oxidation of W-11.4Cr-0.6Y alloy and should be considered in case of its possible application in fusion reactor walls.

Although a complex oxide scale with mixed oxides is formed, the presence of Y seems to play a crucial role in enabling the formation of a continuous, thin chromia scale in the ternary alloy, which can then act as a protective, passivating oxide layer, reducing the rapid formation of mixed oxides and preventing the possible sublimation of the underlying tungsten oxide. In the case of the binary W-Cr alloy, the presence of small chromia inclusions in the outermost oxide layer suggests that some chromia does start to form but is unable to form a continuous layer and, eventually, the formation of a much thicker Cr_2_WO_6_ layer dominates. From the TGA data of the binary alloy, we see that such a mixed oxide configuration grows rapidly and does not show a passivating behavior. The predominant growth of the oxide emerges at the edges and corners, i.e., in the regions with high local curvature, potentially due to higher local stresses in these regions. The oxidation behavior of the binary alloy seems to resemble that of pure W, which shows a tendency to oxidize in this fashion [41]. It should be noted that the thickness of the entire oxide scale in the flat surface of the binary sample is only slightly more than that in the ternary alloy, as seen in Figure 4b,e. Although the Cr_2_WO_6_ layer formed is relatively stable up to 1100 °C [42] and can provide some protection against the sublimation of the tungsten oxide below [7], any parts of the material with high local curvature seem to be potentially weak regions, where the oxide can easily spall off and expose the underlying tungsten oxide to the atmosphere. Due to the significant edge effect seen in the oxidation of the binary alloy, the oxidation rate can also be expected to be different from the measured value of 5.64 × 10^−4^ mg·cm^−2^·s^−1^ in this study if a larger sample or a different geometry is used. The addition of Y also seems to eliminate this edge effect by enabling the formation of a continuous chromia layer.

Apart from the sublimation of tungsten oxide, which should be avoided, it is also well known that at temperatures ≥ 1000 °C, in the presence of water vapor, chromia can form volatile hydroxides [43,44]. Y addition could have an effect on this as well since it results in the formation of large W-Y-O oxide particles on the surface, even after 8 h of oxidation, which could suppress this volatilization of chromia and increase the stability of the chromia layer, especially in humid environments. The stability of the inner layers of the oxide scale is also important to consider when looking at the adherence of the oxide scale to the underlying material. A porous tungsten oxide layer is formed in both cases, but with the addition of Y, the oxide scale shows lower porosity, which could be beneficial for the adherence of the oxide scale to the underlying alloy.

The observed benefits of adding Y to the W-Cr system seem to align with the proposed mechanisms of the reactive element effect of Y on chromia scales, such as the increased adherence of the oxide scale due to a reduction in porosity and a marked reduction in the oxide growth rate and thickness, enabling the growth of chromia to dominate and form a protective layer. These effects are assigned to the segregation of Y at oxide GBs [23]. However, in order to study the exact mechanism of this effect in the W-Cr system, nanoscale analysis of the oxide and alloy GBs and interfaces is necessary. An ongoing study involving the use of high-resolution techniques, such as atom probe tomography, which can provide such information, aims to address these aspects and will be published separately.

## 5. Conclusions

In this work, the effect of Y addition on the microstructure and oxidation resistance of W-Cr at 1000 °C is analyzed, which is a potential plasma-facing material for the first wall of a future fusion reactor. The addition of Y results in a great improvement in oxidation resistance:

1. The rate of oxidation of the ternary W-11.4Cr-0.6Y alloy is reduced 50-fold as compared to the binary W-11.4Cr, with no indication of spalling off. The oxide formed does not show a large volume expansion, which results in the material being able to retain its geometric integrity even after oxidation for 20 h.
2. The formation of a complex oxide scale with various mixed oxides occurs in both cases. However, only the ternary alloy shows the formation of a continuous, thin chromia scale above the mixed oxide, which plays the role of a protective, passivating oxide layer. In the case of the binary alloy, the formation of a much thicker Cr_2_WO_6_ layer dominates, which grows rapidly, mainly at the regions of high local curvature, such as sample edges and corners, and does not become passivating.
3. Although a tungsten oxide layer is formed in both alloys, the oxide scale in the ternary alloy is less porous and is more adherent to the alloy, making it more effective at preventing the possible sublimation of the underlying tungsten oxide by making the oxide scale more resistant to spallation.

## Data Availability

The original contributions presented in the study are included in the article/Supplementary Material; further inquiries can be directed to the corresponding author/s.

## Supplementary Materials

The following supporting information can be downloaded at https://www.mdpi.com/article/10.3390/ma17235749/s1, Figure S1: Large-area SEM images of the cross-section of the oxide scale and the underlying alloy, showing the presence of internal oxidation in (a) the binary alloy and (b) the ternary alloy; Figure S2: W-Cr binary phase diagram [45] with the alloy composition and sintering temperature marked; Table S1: Summary of microstructures of oxidized ternary W-11.4Cr-0.6Y samples from different batches.
