# Supplementary material for: The Effect of Y Addition on Oxidation Resistance of Bulk W-Cr Alloys
Source: Materials (Basel). 2024 Nov 24;17(23):5749. doi: 10.3390/ma17235749 (PMC11642325; doi:10.3390/ma17235749)
Supplement: Supplementary file 1 [file materials-17-05749-s001.zip › materials-3280904-supplementary.pdf]

Supplementary information

Table S1: Summary of microstructures of oxidized ternary W-11.4Cr-0.6Y samples from different batches. Mixed oxide microstructures are marked with a blue box.

	Ingot number, as-sintered microstructure	Temperature	Time	Atmosphere	Sample number, cross-section
1	FAST2060 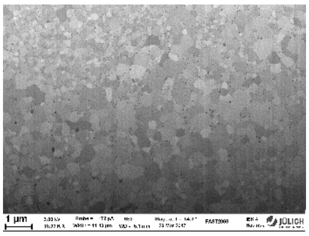 (bimodal grain structure)	1000 °C	44 h	Synthetic air, 70% RH	03 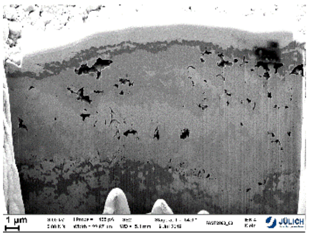
2	FAST2061 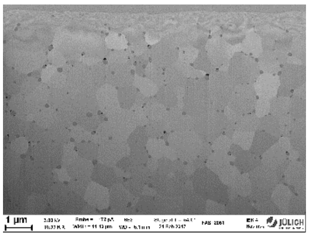 (large (~1 µm) grain size)	1000 °C	44 h	Ar-O_2, dry	04 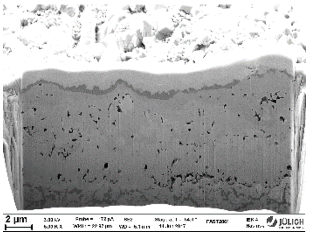
3	FAST2063 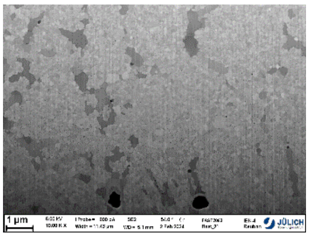	1000 °C	20 h	Synthetic air, 70% RH	07 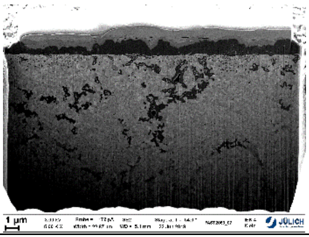
		1000 °C	20 h	Ar-O_2, dry	09 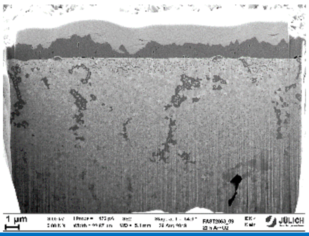
		1000 °C	8 h	Ar-O_2, dry	Rest-01 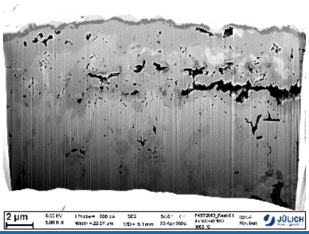

	Ingot number, as-sintered microstructure	Temperature	Time	Atmosphere	Sample number, cross-section
4	FAST2064 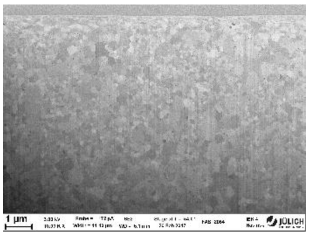	1000 °C	44 h	Ar-O_2, dry	01 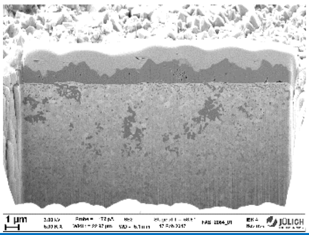
		1000 °C	3 h	Ar-O_2, dry	05 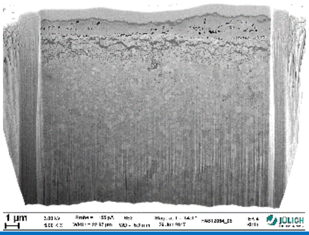
5	FAST4043 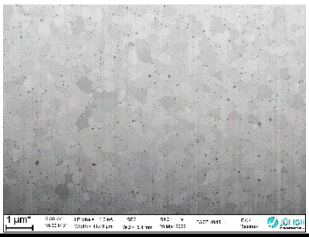	1000 °C	8 h	Ar-O_2, dry	Flat-01 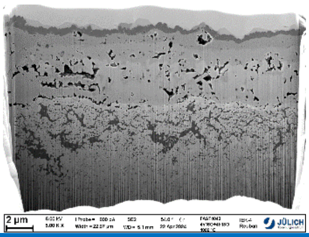
6	FAST4608 (this work) 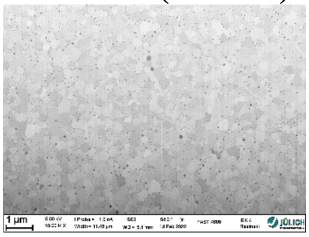	1000 °C	8 h	Ar-O_2, dry	Flat-01 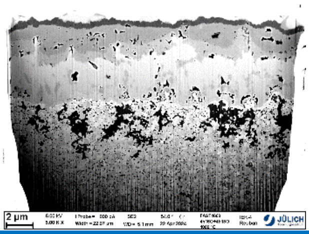
		1000 °C	20 h	Synthetic air, 70% RH	05 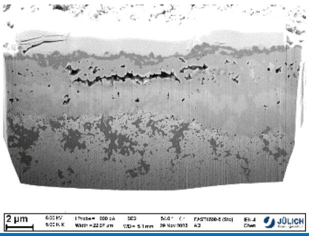

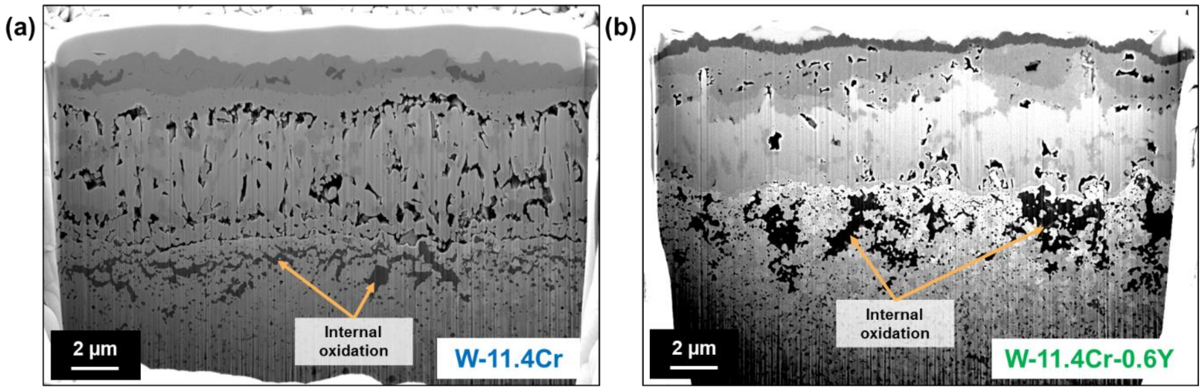

Figure S1: Large-area SEM images of the cross-section of the oxide scale and the underlying alloy, showing the presence of internal oxidation in (a) the binary alloy and (b) the ternary alloy.

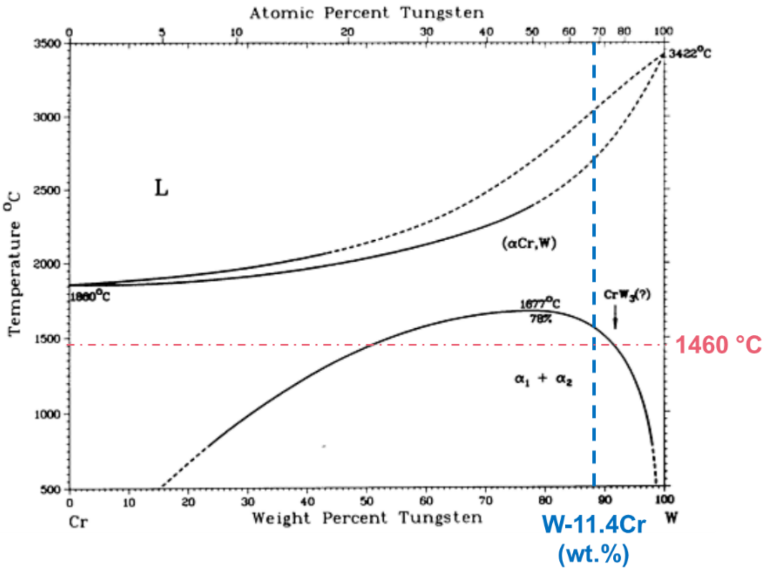

Figure S2: W-Cr binary phase diagram [45] with the alloy composition and sintering temperature marked.
